# Efficacy and safety of selective serotonin reuptake inhibitors for diabetes mellitus with depression: a systematic review and meta-analysis

**DOI:** 10.3389/fendo.2026.1891729

**Published:** 2026-07-22

**Authors:** Yuqing Zhang, Shilin Liu, Andong Li, Zheng Nan

**Affiliations:** 1 Changchun University of Chinese Medicine, Changchun, China; 2 Affiliated Hospital of Changchun University of Chinese Medicine, Changchun, China

**Keywords:** clinical efficacy, depression, diabetes, selective serotonin reuptake inhibitors (SSRIs), system evaluation

## Abstract

**Objectives:**

To evaluate the efficacy and safety of selective serotonin reuptake inhibitors (SSRIs) in patients with diabetes mellitus and comorbid depression.

**Methods:**

This study was registered in PROSPERO (CRD420261305439). Eight electronic databases were searched up to February 11, 2026 for randomized controlled trials comparing SSRIs plus usual care versus control. Outcomes included depressive symptoms (HDRS, BDI), anxiety (HARS), glycaemic control (HbA1c, FPG, PPG), and BMI. Meta-analyses were performed using random-effects models; subgroup analysis by follow-up duration and sensitivity analysis were conducted.

**Results:**

Nine RCTs (770 participants) were included. Overall, SSRIs did not significantly improve HDRS (MD = 0.16, 95% CI -0.69 to 1.01, *P = 0.72*) or BDI scores. However, subgroup analysis showed significant HDRS reduction when follow-up was ≥ 6 months (MD = -0.96, 95% CI -1.86 to -0.05, *P = 0.04*). SSRIs were associated with increased HARS scores (MD = 2.37, 95% CI 0.73 to 4.02, *P = 0.005*). No significant effects were observed for HbA1c, FPG, PPG, or BMI.

**Conclusion:**

Preliminary evidence suggests that SSRIs may offer modest antidepressant benefits when used for ≥ 6 months in diabetic patients with depression, but they may be associated with a small increase in anxiety scores and do not appear to improve glycaemic control or BMI. However, the overall certainty of evidence is low to moderate. Monitoring of early anxiety symptoms is recommended, and further high-quality RCTs are needed.

**Systematic Review Registration:**

https://www.crd.york.ac.uk/prospero/, identifier CRD420261305439.

## 1 Introduction

Diabetes is a very common chronic metabolic disease in the world, and its prevalence is increasing, which has become a serious public health problem (1). Diabetes not only affects the disorder of blood glucose control and metabolic balance, but also is often accompanied by significant mental and psychological comorbidities (2). Among these psychological problems, depression is the most common mental complication in patients with diabetes (3). According to epidemiological data, the risk of depression in diabetic patients is 2–3 times higher than that in non-diabetic patients. About 20%–30% of diabetic patients have comorbid depression, which is much higher than 5%–10% of the general population (4). This situation of diabetes combined with depression is not accidental (5):on the one hand, diabetic patients are prone to induce or aggravate depression when they face the pressure of disease management, the potential threat of complications and many restrictions of lifestyle for a long time (6); on the other hand, depression further deteriorates blood glucose control and increases the risk of diabetes-related complications and mortality by activating the hypothalamus-pituitary-adrenal axis, promoting the release of inflammatory factors, and reducing treatment compliance (7). Diabetes and depression interact with each other and form a vicious circle, which not only greatly reduces the quality of life of patients, but also brings a heavy burden to society and economy (8).

At present, the common treatment mode of diabetes mellitus complicated with depression is “hierarchical management”: hypoglycemic drugs to control blood glucose, antidepressants or psychotherapy to deal with emotional problems (9). However, in the actual clinical treatment process, there are many problems. First of all, some hypoglycemic drugs (10) (such as insulin, sulfonylureas) may cause patients to have a fear of hypoglycemia, which in turn indirectly aggravates anxiety and depression. Secondly, the use of traditional antidepressants (11) (such as tricyclic antidepressants) in patients with diabetes has been limited due to adverse reactions such as weight gain, increased insulin resistance, and arrhythmia. Third, although psychotherapy (12) (such as cognitive behavioral therapy) has a certain effect, it is difficult to popularize and apply in a wider range due to the difficulty of obtaining professional psychotherapy resources, long treatment cycle and relatively high treatment cost.

Selective serotonin reuptake inhibitors (SSRIs) are the current first-line antidepressants, which exert antidepressant effects by blocking 5-HT reuptake and enhancing central 5-HT neurotransmission (13). Compared with traditional antidepressants, SSRIs have better safety and tolerance: less sedative and anticholinergic adverse reactions (dry mouth, constipation, blurred vision), little effect on cardiac conduction, and relatively high safety in the case of drug overdose (14). For diabetic patients with depression, SSRIs may have some unique theoretical advantages: First, don’t increase the risk of hypoglycemia (most studies did not report severe hypoglycemia events); Second, some SSRIs (such as sertraline and fluoxetine) had neutral or mild beneficial effects on body weight and glucose metabolism. Third, it can improve the physical symptoms associated with diabetes (such as fatigue, appetite changes, sleep disorders) (15, 16). However, the existing clinical studies on the application of SSRIs in diabetic patients with depression have some limitations. The sample size of these studies is generally small, the follow-up time is short, the outcome indicators used are different, and the impact on patients’ anxiety symptoms and metabolic indicators is not fully reported. These factors make the efficacy and safety of SSRIs in this special patient population still controversial.

Up to now, there is no systematic review or meta-analysis that comprehensively integrates the efficacy and safety of SSRIs in the treatment of diabetes mellitus complicated with depression, especially the lack of simultaneous evaluation. The results of this study will provide evidence-based basis for clinicians to rationally use SSRIs in patients with diabetes and depression, and provide optimization suggestions for future clinical trial design.

## 2 Methods

This study was conducted according to the Cochrane Handbook for Systematic Reviews of Interventions, PRISMA 2020 Checklist (**Supplementary S1**). In addition, this review is registered in PROSPERO (https://www.crd.york.ac.uk/PROSPERO/view/CRD420261305439).

### 2.1 Search strategies

We searched eight electronic databases, including PubMed, Cochrane Library, Embase, Web of Science, China National Knowledge Infrastructure (CKNI), Wanfang Database, Scopus, Chinese Biomedical Medicine Database (CBM), and Weipu Information Resource Integration Service Platform. In addition, the Chinese Clinical Trial Registry (CHICCTR) has also been searched for ongoing or completed but unpublished randomized controlled trials (RCTs). The databases were independently searched from their respective inception dates until February 11, 2026. We included all randomized controlled trials of selective serotonin reuptake inhibitors (SSRIs) in the treatment of depression in diabetic patients. The search terms included “selective serotonin reuptake inhibitor”, “selective serotonin reuptake inhibitor”, “fluoxetine”, “paroxetine”, “sertraline” “diabetes”, “depression” etc. A comprehensive search strategy for these databases is shown in the supplementary file (**Supplementary S2**). There are no restrictions on language.

### 2.2 Inclusion and exclusion criteria

All randomized controlled trials evaluating selective serotonin reuptake inhibitors in the treatment of diabetes mellitus with depression were included in the meta-analysis. Inclusion criteria are as follows: 1) The subjects met the World Health Organization diagnostic criteria for diabetes with depression, and the patient’s age, gender, race, course of disease, and education level were not restricted; 2) The study type was limited to RCT. Exclusion criteria are as follows: 1) studies involving combination regimens, animal experiment, review, meta-analysis; 2) Data missing, incomplete or unable to extract; 3) Patients with significant risk of suicide or comorbidity with other mental diseases; 4) Patients had a history of drug allergy; 5) Repeated publication. Preclinical studies and animal experiments were excluded because the primary aim of this systematic review was to evaluate clinical efficacy and safety outcomes in human populations to inform clinical practice. While animal models can provide mechanistic insights, they are not directly generalisable to human patients and cannot be pooled with clinical data in a meta−analysis. Therefore, only human RCTs were considered for inclusion.

### 2.3 Research selection and data extraction

Three researchers (Z-YQ, L-SL, L-AD) searched and screened suitable studies according to pre-determined inclusion and exclusion criteria. In the case of differences, the final decision is made by consensus. In order to manage the literature, Endnote 21 software was used. Two evaluators (Z-YQ and L-SL) independently extracted relevant data from eligible studies using standardized excerpt forms, including: first author, year of publication, sample size, average age, gender, course of disease, intervention measures, and outcome indicators. The extracted data is cross-checked by (Z-YQ) and (L-SL), and a third reviewer (L-AD) is available to resolve any conflicts.

### 2.4 Risk of bias assessment

Two reviewers (Z-YQ and L-SL) independently assessed the risk of bias based on Review Manager 5.4 ‘s bias risk tool, which includes the following criteria: random sequence generation, allocation concealment, blinding of participants and personnel, blinding of outcome assessments, incomplete outcome data, selective reporting, and others. The results were judged to be “low”, “high” or “unclear”, and any disagreement was resolved by a third investigator (L-AD).

### 2.5 Data synthesis and statistical analysis

Review Manager 5.4 was used to analyze and evaluate the effect of selective serotonin reuptake inhibitors (SSRIs) on diabetes with depression. Weighted mean difference (WMD) and *95% confidence interval* (*95% CI*) were used to evaluate various depression scales, HbA1c, FPG and BMI. The heterogeneity of the data was examined by the X^2^ test and the I^2^ test. The X² test assesses whether the observed variation between study results is greater than expected by chance alone; P < 0.05 indicates significant heterogeneity and is considered statistically significant. The *I^2^* test quantifies the proportion of total variation that is due to heterogeneity rather than chance, with values of 25%, 50%, and 75% representing low, moderate, and high heterogeneity, respectively. If heterogeneity was low (*P > 0.05*, *I^2^* < 50%), a fixed-effect model was used; otherwise, a random-effects model was applied. In order to explore the potential sources of heterogeneity, we analyzed the factors leading to heterogeneity through subgroup analysis. In addition, due to the final screening of less than 10 articles, no funnel plot was performed to assess publication bias.

### 2.6 Sensitivity analysis

In order to evaluate the robustness and reliability of the comprehensive results in meta-analysis, we use sensitivity analysis as an important method. Sensitivity analysis was performed by excluding individual studies in turn and conducting a meta-analysis of the remaining studies. We evaluated whether the obtained results were significantly different from the results before exclusion to ensure the robustness of the results.

### 2.7 Subgroup analysis

In order to explore the effect of different follow-up time on the intervention effect, this study conducted a subgroup analysis of the main outcome indicators. According to the follow-up duration of each study, the included data sets were divided into two subgroups: short-term follow-up (< 6 months) and long-term follow-up (≥ 6 months). The combined effect size in each subgroup was calculated respectively, and the heterogeneity test (*I^2^* statistics) between subgroups was used to evaluate whether the follow-up time had a significant impact on the combined results. This subgroup analysis is helpful to determine the potential differences in depressive symptoms and blood glucose control in different treatment cycles of antidepressant treatment, and to provide a more detailed reference for clinical practice.

## 3 Results

### 3.1 Literature selection

**Figure 1:**
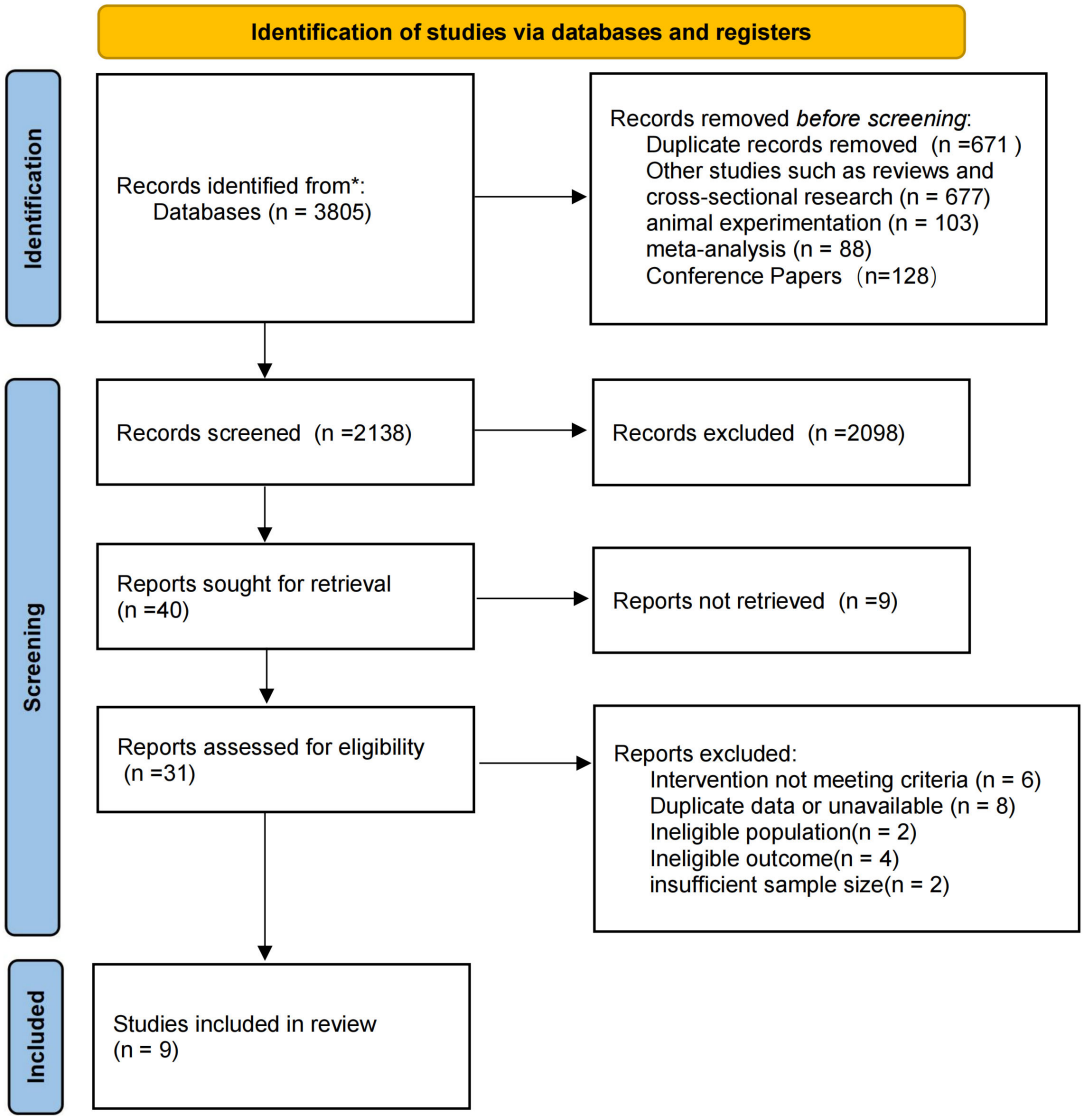
Flow diagram of studies selection process.

Through our search strategy, we preliminarily evaluated 3805 related articles. After removing duplicates, the remaining 3134 articles were screened by title and abstract, of which 996 articles needed further screening after excluding reviews, animal experiments and other articles that did not meet the inclusion criteria. After carefully reading the full text of 31 articles, 22 articles were excluded for the following reasons: Intervention not meeting criteria (n=6), Duplicate data or unavailable (n=8), Ineligible population (n=2), Ineligible outcome (n= 4), Insufficient sample size (n=2). Finally, 9 articles (Lustman et al., 2000 (17); Williams et al., 2007 (18); Karaiskos et al., 2013 (19); Kang et al., 2015 (20); Petrak et al., 2015 (21);Kumar et al., 2015 (22); Che et al., 2018 (23); Kumar et al., 2019 (24); Nicolau et al., 2013 (25)) were included in this meta-analysis. The study selection process is shown in **Figure 1**.

### 3.2 Study characteristics

A total of 9 studies involving 770 patients were included, including 403 in the treatment group and 367 in the control group. All studies were randomized controlled trials conducted between 2000 and 2019. In these trials, the intervention in the treatment group was the addition of SSRIs based on hypoglycemic therapy, while there were three interventions in the control group: three studies (17, 18, 25) used placebo, 4 studies (19, 20, 22, 23) used agomelatine, one study (21) used Cognitive Behavioral Therapy, and one study (24) only used hypoglycemic therapy. Among the nine randomized controlled trials, there were two studies in the treatment group (17, 23) took fluoxetine, 4 studies (18, 19, 21, 24) took sertraline, one study took paroxetine in 20, one study took escitalopram in 22, and one study took citalopram in 25. The shortest and longest intervention time was 1 month and 13 months, respectively. In the end, only 9 randomized controlled trials met the inclusion criteria, but when reading the full text to extract data, a total of 14 independent data were compiled. This is because part of the study (18, 20–22, 24) contains multiple comparable groups or subgroups that can be independently analyzed, such as the results of different age groups, different antidepressants or different follow-up time points, and each group is considered as an independent data unit for quantitative synthesis. This method enables us to make full use of existing evidence while ensuring the integrity of meta-analysis. For the nine included studies, **Table 1** presents the characteristics of 14 datas. To improve transparency, we further extracted detailed information on patient characteristics (diabetes duration, baseline HbA1c, baseline depression severity), SSRI regimens (type, dose range, titration schedule), and dropout rates per study. These data are summarized in **Table 1**. In brief, the mean diabetes duration across studies ranged from 6.5 to 12.3 years, and baseline HDRS scores ranged from 12 to 22. The most commonly used SSRIs were sertraline (50–150 mg/day) and fluoxetine (20–40 mg/day). Dropout rates varied from 6% to 28%, with the most common reasons being withdrawal of consent and loss to follow-up. No study reported a dropout rate exceeding 30%.

**Table 1:** Characteristics of the included studies.

Study	Samplesize(T/C)	Age (Y)		Gender(M/F)		Educational status		Intervention		Duration(MON)	Outcome
		T	C	T	C	T	C	T	C		
Lustman et al. (17)	27/27	45 + 13	47.7 + 11.5	5/22	11/16	14.3 + 2	14.4 + 2.2	Fluoxetine	placebo	2	(3)(5)
Williams et al. (18)	47/38	43 + 8.1	45.3 + 7.1	20/27	15/23	14.2 + 2.6	14.5 + 2.1	Sertraline	placebo	13	(1)(3)(5)
Williams et al. (18)	32/35	61.5 + 6.2	66.3 + 6.2	13/19	13/22	14.2 + 2.8	13.5 + 2.9	Sertraline	placebo	13	(1)(3)(5)
Karaiskos et al. (19)	20/20	52.4 + 11.4	54.3 + 12.5	NR	NR	9.6 + 3.6	9.1 + 4.1	Sertraline	agomelatine	4	(1)(2)(3)(4)
Kang et al. (20)	56/60	52.5 + 10.3	50.8 + 11.4	31/25	32/28	9.67 + 5.33	9.53 + 4.79	Paroxetine	agomelatine	3	(1)(2)(3)(4)(5)(6)
Kang et al. (20)	56/60	52.5 + 10.3	50.8 + 11.4	31/25	32/28	9.67 + 5.33	9.53 + 4.79	Paroxetine	agomelatine	1.5	(3)(4)
Petrak et al. (21)	62/53							Sertraline	CBT	15	(1)(3)
Petrak et al. (21)	62/53							Sertraline	CBT	3	(1)(3)
Kumar et al. (22)	20/20	48.65 + 10.19	49.75 + 14.27	NR	NR			Escitalopram	agomelatine	2	(1)(2)(3)(5)
Kumar et al. (22)	20/20	48.65 + 10.19	49.75 + 14.27	NR	NR			Escitalopram	agomelatine	1	(1)(2)(5)
Che et al. (23)	36/41	52.65 + 10.77	51.66 + 10.3	25/15	23/21	9.18 + 3.12	9.36 + 3.1	Fluoxetine	agomelatine	3	(2)(1)(2)(3)(4)(6)
Kumar et al. (24)	65/63	49.12 + 1.04	50.02 + 1.74	44/21	NR			Sertraline	hypoglycemic therapy only	6	(1)(2)(5)
Kumar et al. (24)	65/63	49.12 + 1.04	50.02 + 1.74	44/21	NR			Sertraline	hypoglycemic therapy only	3	(1)(2)(5)
Nicolau et al (25)	38/10	60.35 + 10.73	59.4 + 11.61	19/19	NR	30/61/9	40/60/0	Citalopram	placebo	6	(1)(5)(6)

### 3.3 Risk of bias assessment

**Figure 2:**
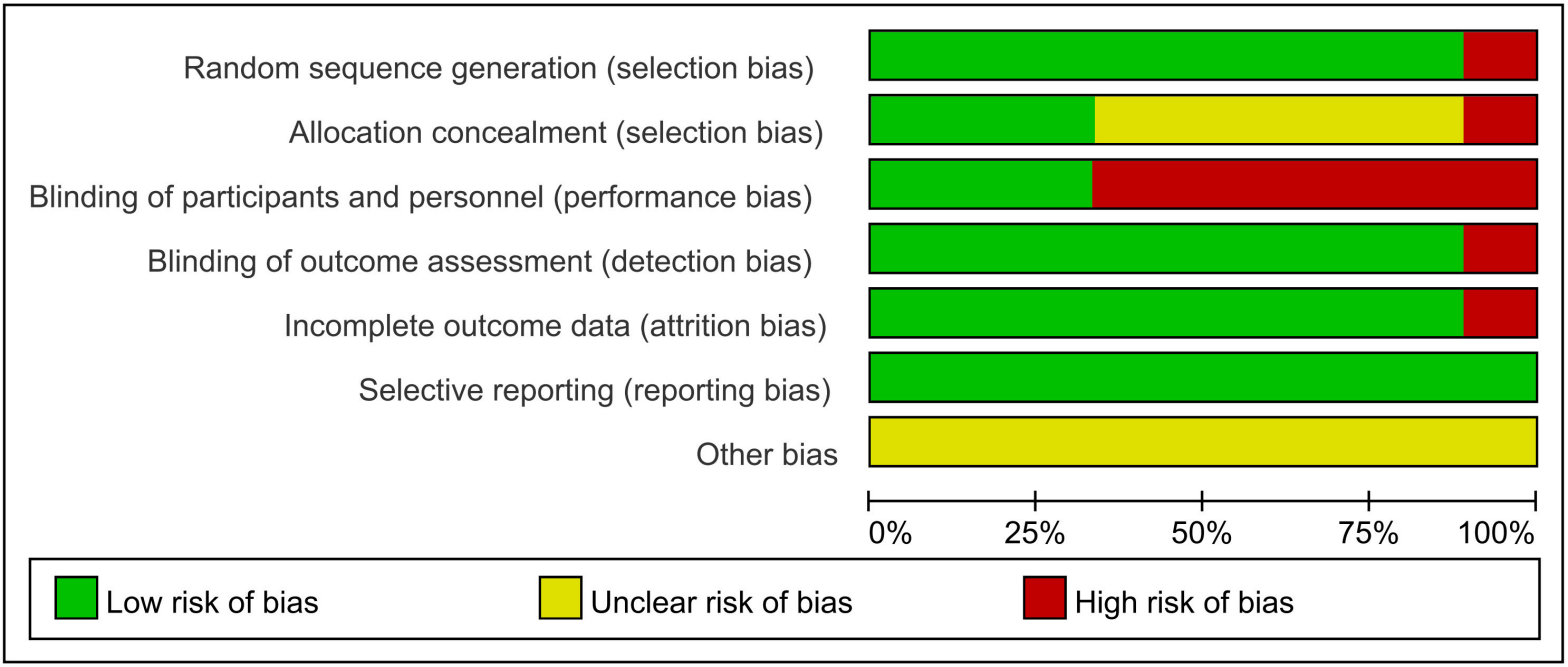
Risk of bias graph.

**Figure 3:**
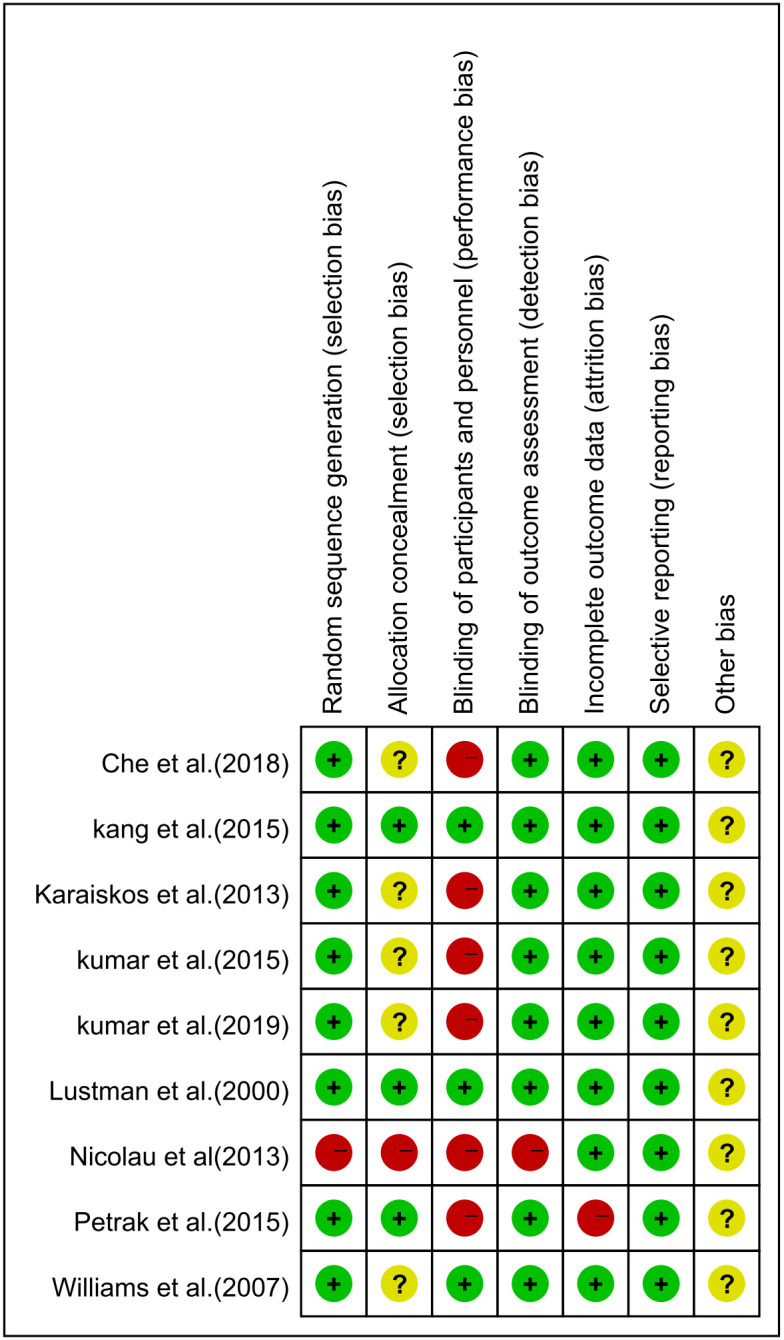
Risk of bias summary.

Bias risk assessment results **Figure 2** and **Figure 3** are shown. In the 9 included studies, a total of 3 studies (17, 18, 20) were judged to have a low risk of bias due to the use of double-blind design, computer-generated random sequences and well-hidden allocation (while the outcome data was complete). A total of 5 studies (19, 21–24) although randomization (computer-generated or central randomization) was reported, it was judged to have a high risk of bias due to the use of open label or single-blind design (participants or researchers were not blinded), and some studies 21 had a high long-term follow-up exit rate. 25 did not use randomization (patients with depression choose whether to receive treatment), and there was a serious selection bias, so it was also judged as a high risk of bias. In terms of allocation concealment, except 17, 20, 21 clearly described the allocation concealment method, and the remaining studies did not report the allocation concealment details, so they were labeled as “unclear risk”. All nine studies did not implement a completely blind method (open label or single-blind design) for participants and researchers, and were therefore judged to have a high risk of bias in this area. However, the main outcome indicators (HbA1c, blood glucose, etc.) of these studies are objective laboratory test results, and the depression assessment uses a standardized scale, so the risk of outcome measurement bias is low. In terms of other biases, all studies did not provide sufficient information to determine whether there were other significant biases, so they were assessed as “unclear risk”.

### 3.4 Outcomes

#### 3.4.1 Effect of SSRIs on depression scale indicators

##### 3.4.1.1 Hamilton Depression Rating Scale

**Figure 4:**
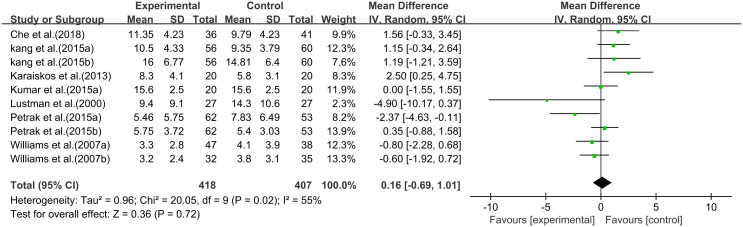
Forest plot for HDRS.

A total of 10 studies (about 825 patients) (17–23, 26) used the HDRS to evaluate the degree of depression in patients. There was moderate heterogeneity among the studies *(I^2^ = 55%, P = 0.02*), so the random effect model was used for combined analysis. The combined results showed that there was no significant difference in HDRS scores between the experimental group and the control group after treatment (combined MD = 0.16, 95% CI [-0.69,1.01], *Z = 0.36, P = 0.72*). The forest map (**Figure 4**) intuitively showed the effect size and combined results of each study. The 95% confidence interval of all studies crossed the invalid line (0), and the combined diamond intersected with the invalid line, suggesting that the improvement effect of this intervention on depressive symptoms was not better than that of the control.

**Figure 5:**
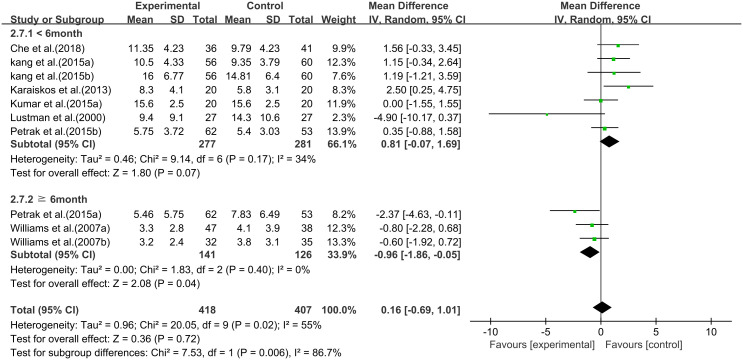
Forest plot for HDRS subgroup analysis.

Subgroup analysis (**Figure 5**) was performed according to the follow-up time. Subgroup analysis was performed according to the follow-up time (< 6 months and ≥ 6 months). Seven studies (n=556) with follow-up time < 6 months and three studies (n=443) with follow-up time ≥ 6 months were included. For studies with a follow-up time of < 6 months, the experimental group and the control group (combined MD = 0.81, 95% CI [-0.07, 1.69], *Z = 1.80, P = 0.07; I^2^ = 34%*, the difference of heterogeneity test (*P < 0.17*) was not statistically significant. For studies with a follow-up time of ≥ 6 months, the HDRS score of the experimental group was significantly lower than that of the control group (combined MD = -0.96, 95% CI [-1.86, -0.05] *Z = 2.08, P = 0.04; I^2^ = 0%*, heterogeneity test *P = 0.04*). Subgroup difference test confirmed (Chi^2^ = 7.53, df = 1, *P = 0.006; I^2^ = 86.7%*), indicating that the follow-up time significantly affected the treatment effect.

**Figure 6:**
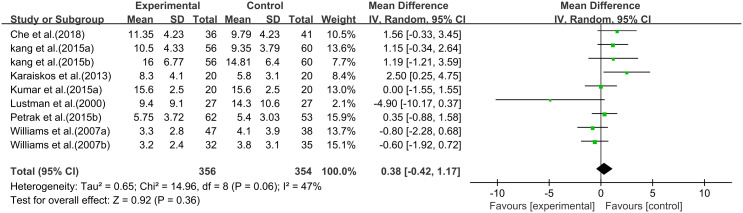
Forest plot for HDRS sensitivity analysis.

In order to evaluate the impact of a single study on the combined results, we performed a sensitivity analysis (**Figure 6**) by eliminating the method one by one. The combined results after excluding 21 are shown here (the weight of this study in the total analysis is 8.2%, and the effect direction is different from other studies). After excluding this study, a total of 9 studies (n = 710) were included. The heterogeneity was reduced (*I^2^ = 47%, P = 0.06*), suggesting that the study may be one of the sources of heterogeneity. The combined effect size still showed no statistically significant difference in HDRS scores between the experimental group and the control group (*Z = 0.92, P = 0.36*), which was consistent with the overall analysis results (*P = 0.72*). This shows that the overall analysis results are relatively robust and are not subject to the dominant influence of this single study.

##### 3.4.1.2 Hamilton Anxiety Rating Scale

**Figure 7:**
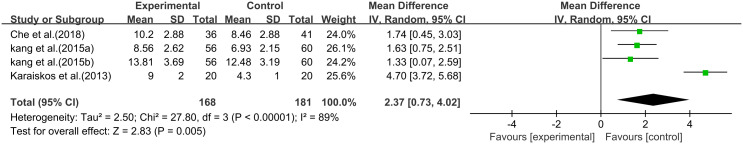
Forest plot for HARS.

This analysis included 4 studies (349 patients), (19, 20, 23) evaluated the effect of experimental measures on the HARS score. (**Figure 7**) The combined results showed that the HARS score of the experimental group was significantly higher than that of the control group, and the difference was statistically significant (combined effect size MD = 2.37, 95% CI [0.73, 4.02], *Z = 2.83, P = 0.005*). All the included studies showed the effect direction favor control group, indicating that the experimental measures may be related to the aggravation of anxiety symptoms. However, there was significant heterogeneity between studies (*I^2^ = 89%, P < 0.00001*), and all studies had a high risk of bias in blinding. Therefore, this finding needs to be interpreted cautiously. At present, the evidence cannot draw a definitive conclusion that experimental measures lead to increased anxiety.

**Figure 8:**
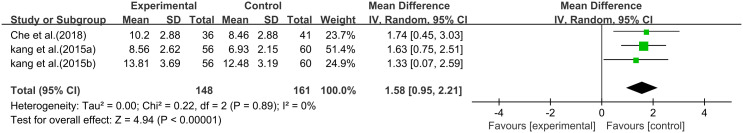
Forest plot for HARS sensitivity analysis.

Based on the sensitivity analysis of HARS indicators (**Figure 8**). After excluding the 19 study, a total of 3 studies were included, totaling 309 patients. The combined results showed that the HARS score of the experimental group was still significantly higher than that of the control group, and the difference was statistically significant (combined effect size MD = 2.58, 95% CI [0.95, 2.21], *Z = 4.94, P < 0.00001*). It is worth noting that after excluding this study, the heterogeneity between studies decreased from the original height (*I^2^ = 89%*) to a negligible level (*I^2^ = 0%, P = 0.89*), indicating that the study of 19 may be the main source of heterogeneity in the original analysis. The sensitivity analysis results were consistent with the original combined results (MD = 2.37) and the effect size was similar, which confirmed the robustness of the HARS analysis results. Therefore, although the original analysis suggested that experimental measures may be associated with elevated HARS scores, this finding is more reliable based on the consistency and low heterogeneity of sensitivity analysis. However, all included studies still have a high risk of bias in blinding, the conclusions still need to be interpreted carefully, and more high-quality randomized controlled trials are expected to be verified.

##### 3.4.1.3 Beck Depression Inventory

**Figure 9:**
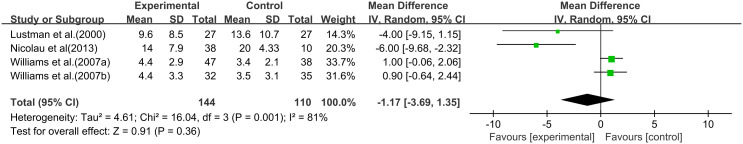
Forest plot for BDI.

A total of 4 studies were included, including 254 subjects. Heterogeneity test showed that there was a high degree of heterogeneity between studies (*I^2^ = 81%, P = 0.001*), so a random effect model was used. The combined effect size MD = -1.17, 95% CI [-3,69,1.35], *Z = 0.91, P = 0.36*). The overall effect test showed that there was no significant difference between the experimental group and the control group. The forest plot (**Figure 9**)showed that the effects of 17 and 25 tended to be beneficial to the experimental group (MD was negative), while the effects of Williams et al. (18) tended to be beneficial to the control group (MD was positive), and inconsistent directions may be the main source of heterogeneity. Current evidence shows that the improvement effect of intervention measures on BDI score is not better than that of the control.

#### 3.4.2 Effects of SSRIs on blood glucose

##### 3.4.2.1 Glycosylated hemoglobin (HbA1c)

**Figure 10:**
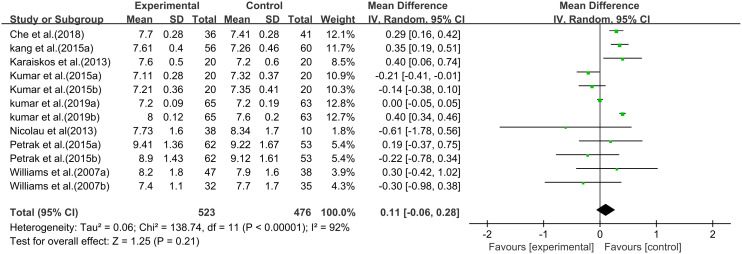
Forest plot for HbA1c.

A total of 12 studies (about 1046 patients) (18–25) evaluated the effect of experimental measures on glycosylated hemoglobin (HbA1c). The combined effect size (**Figure 10**) showed that there was no significant difference between the experimental group and the control group (combined effect size MD = 0.11, 95% CI [-0.06, 0.28], *Z = 1.25, P = 0.21*). Although the combined results were not statistically significant, there was a high degree of heterogeneity among the studies (*I^2^ = 92%, P<0.00001*). This heterogeneity may be due to differences in interventions and control treatments in different studies.

**Figure 11:**
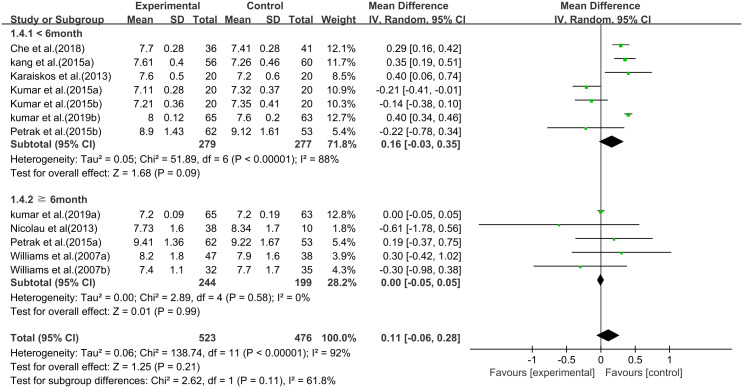
Forest plot for HbA1c subgroup analysis.

Subgroup analysis (**Figure 11**) was stratified by follow-up duration: < 6 months subgroup (7 studies, n = 556) still had high heterogeneity (*I^2^ = 88%*), (the combined MD effect was 0.16 (95% CI: -0.03,0.35), and the difference was not significant (*P = 0.09*); there was no heterogeneity (*I^2^ = 0%*) in the subgroup of ≥ 6 months (5 studies, n = 443), and the combined MD was 0.00 (95% CI: -0.05,0.05), with no significant difference (*P=0.99*). The difference test between subgroups was not significant (*χ^2^ = 2.62, P = 0.11, I^2^ = 61.8%*). Current evidence shows that interventions are not superior to controls in improving HbA1c levels, and the follow-up duration does not show a clear modification effect. Subgroup analysis failed to fully explain the source of heterogeneity. Therefore, the current evidence does not determine the clear effect of experimental measures on the long-term glycemic control index HbA1c.

##### 3.4.2.2 Fasting plasma glucose

**Figure 12:**
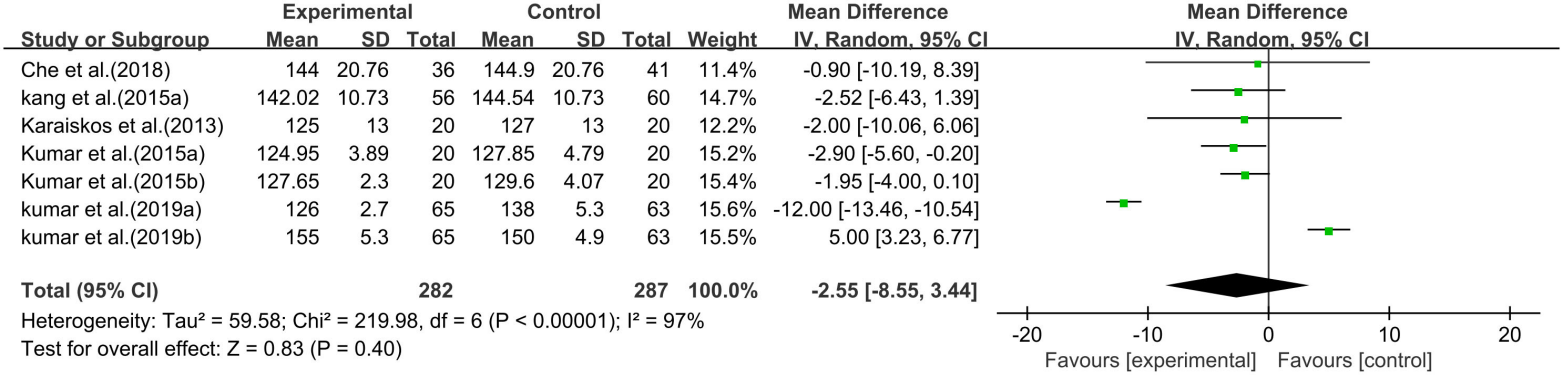
Forest plot for FPG.

7 studies (569 patients) were included (19, 20, 22–24) (**Figure 12**) showed that there was no significant difference in the overall effect of reducing FPG level between the experimental group and the control group (combined effect size MD = -2.55, 95% CI [-8.55, 3.44], *Z = 0.83, P = 0.40*). There was a high degree of heterogeneity among the studies (*I^2^ = 97%, P < 0.00001*). It is worth noting that although most studies estimate the favor experimental group, 24 showed that the effect of the experimental group was significantly worse than that of the control group (MD = 5.00), which may be the main source of heterogeneity. Therefore, there is currently no sufficient evidence that experimental measures are effective in improving FPG, and more well-designed and consistent randomized controlled trials are needed in the future.

##### 3.4.2.3 Postprandial blood glucose

**Figure 13:**
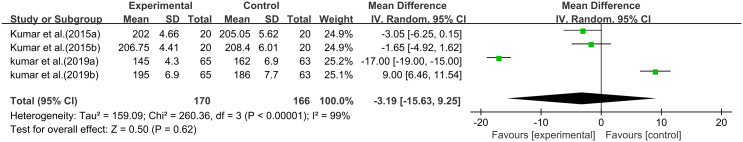
Forest plot for PPG.

Four studies (a total of 336 subjects) were included (22, 22, 24, 24) was designed to evaluate the effect of experimental measures on postprandial blood glucose (PPG). The combined results (**Figure 13**) showed that there was no significant difference between the experimental group and the control group (combined effect size MD = -3.19, 95% CI [-15.63, 9.25], *Z = 0.50, P = 0.62*). However, there was extremely high heterogeneity among studies (*I^2^ = 99%, P < 0.00001*), which may be due to the fact that the results of 24 study (MD = 9.00, favor control) were opposite to the other three studies. In view of the huge inconsistency between studies and the potential limitations of methodological quality, the current evidence does not support that experimental measures are superior to control treatment in reducing PPG, and the results need to be interpreted with extreme caution.

#### 3.4.3 Body mass index

**Figure 14:**
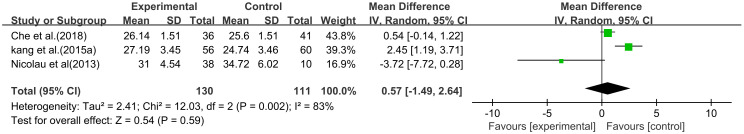
Forest plot for BMI.

Three studies involving 241 patients were included, (20, 23, 25). The results (**Figure 14**) showed that there was no significant difference in BMI changes between the experimental group and the control group (combined effect size MD = 0.57, 95% CI [-1.49, 2.64], *Z = 0.54, P = 0.59*). There was a high degree of heterogeneity among the studies (*I^2^ = 83%, P = 0.002*), which was mainly manifested in 20 study favor control group (MD = 2.45), while 25 study favor experimental group (MD = -3.72). In view of the small number of included studies and the opposite direction of the results, there is no sufficient evidence to support the exact impact of experimental measures on BMI. The conclusion is extremely unstable and needs to be verified by more high-quality studies.

## 4 Discussion

### 4.1 Summary of the main results

A total of 3805 relevant literatures were retrieved in this study, and 9 meta-analysis literatures were included. After reading the full text, 14 independent data were sorted out. The main findings of our meta-analysis are as follows: 1)Depressive symptoms: SSRIs did not significantly improve HDRS or BDI scores in general, but subgroup analysis showed that SSRIs could significantly reduce HDRS scores (MD = -0.96*, P = 0.04*) when the follow-up time was ≥ 6 months, while short-term (< 6 months) was ineffective; 2)Anxiety symptoms: SSRIs were associated with a significant increase in HARS scores (MD = 2.06*, P = 0.005*), suggesting that they may aggravate anxiety. 3) Blood glucose control: SSRIs had no significant effect on HbA1c, fasting blood glucose and postprandial blood glucose. 4)Weight: SSRIs had no significant effect on BMI; 5) Heterogeneity: Most indicators are highly heterogeneous (*I^2^>80%*), mainly from individual studies such as (19, 24).

### 4.2 Long−term antidepressant effect is greater than short−term

This study found that SSRIs had no immediate effect on depressive symptoms in diabetic patients with depression, but mild statistical benefits were observed when the treatment lasted for 6 months or longer This phenomenon may be related to the following mechanisms: 1) It usually takes 4–8 weeks (27) for SSRIs to reach the steady-state plasma concentration and exert a stable antidepressant effect, while patients with diabetic comorbid depression are often accompanied by more complex neuroendocrine disorders (such as excessive activation of HPA axis and elevation of inflammatory factors), which may take longer to achieve clinical response (28). 2) In the short term (< 6 months), the placebo effect, the non-specific effect of psychological support and the patient ‘s expectation of treatment may cover up the real difference. 3) During the long-term follow-up, the patients in the control group may have decreased compliance or accumulated psychological stress caused by hypoglycemic therapy, while the advantages of SSRIs in continuously improving mood gradually emerged. Compared with previous studies, there are few SSRIs trials for patients with diabetes mellitus and depression. However, a meta-analysis showed that the effect size (MD) of antidepressants (mainly SSRIs) on depressive symptoms was about 0.2-0.4, which was basically consistent with the effect size (about 0.2-0.3 standard deviation) of long-term follow-up in this study. However, most previous studies did not stratify according to the length of follow-up, which may mask the time-dependent effect. It is worth noting that the total combined results of the BDI scale (MD = -0.94, *P = 0.36*) were consistent with the HDRS results, which did not support the overall validity. Different depression scales (HDRS-17 vs BDI) may have different construct validity in diabetic population. HDRS focuses more on somatic symptoms (appetite, sleep, fatigue), while BDI emphasizes more on cognitive-emotional dimension. SSRIs may take effect more slowly on somatic symptoms, which may be one of the reasons why long-term follow-up is effective.

### 4.3 SSRIs cause anxiety symptoms

The most surprising finding was that SSRIs treatment was associated with a significant increase in anxiety scores (HARS MD = 2.06, *P = 0.005*), and the conclusion was still robust after excluding 19 (MD = 1.58, *P < 0.0001*), which was contrary to the clinical practice of SSRIs for anxiety disorders (29, 30). Several mechanisms may explain this increase. First, SSRIs can cause activation syndrome in the first 2–4 weeks of treatment, characterized by agitation, restlessness, and heightened anxiety, which is thought to be mediated by overstimulation of 5−HT_2_C receptors in the raphe nuclei and amygdala (31). Second, patients with diabetes often exhibit dysregulation of the hypothalamic−pituitary−adrenal (HPA) axis (32, 33) and elevated baseline inflammatory cytokines, which may lower the threshold for SSRI−induced anxiogenic effects. Third, there may be a direct metabolic interaction: some SSRIs (e.g., paroxetine) have been associated with mild hypoglycemia or weight changes, which could trigger fear of hypoglycemia and consequent anxiety in diabetic patients (34). Beyond these biological mechanisms, other factors may also contribute. For instance, some control groups received psychotherapy (e.g., cognitive−behavioral therapy) known to be effective for anxiety, whereas SSRI monotherapy (35) may be inferior in comparison. Additionally, only four studies reported HARS, and the sample size was small, which cannot rule out chance findings. These mechanisms and factors are not mutually exclusive and warrant further investigation. These clinical observations are partially supported by preclinical evidence. Animal studies have shown that SSRI administration can acutely activate 5−HT2C receptors in the raphe nuclei and amygdala, leading to increased anxiety−like behaviours during the early treatment phase. Although these findings are not directly translatable to humans, they provide plausible mechanistic support for our observed increase in HARS scores.

However, several caveats must be emphasised: only four studies (349 patients) contributed to the HARS analysis, all with high risk of bias (open−label or single−blind), and the heterogeneity was substantial (*I² = 89%*). Sensitivity analysis excluding Karaiskos et al. reduced heterogeneity to 0% but did not change the direction. Therefore, while the finding that SSRIs may worsen anxiety is important, it should be considered hypothesis−generating rather than definitive.

We suggest that clinicians proactively monitor patients’ anxiety symptoms when using SSRIs, especially in the early stage of treatment. If anxiety is aggravated, it may be considered to reduce the dose, switch to antidepressants with weaker activation (such as escitalopram), or combine short−term benzodiazepines to control symptoms. Dedicated RCTs with systematic anxiety monitoring are urgently needed.

### 4.4 No significant improvement in blood glucose control and body weight

This analysis did not find that SSRIs had a consistent improvement effect on HbA1c, fasting blood glucose, postprandial blood glucose and BMI. Although individual studies (such as 24) reported a significant decrease in fasting blood glucose, another study by the same authors (24) showed an increase in fasting blood glucose, with conflicting directions leading to extremely high heterogeneity (*I^2^ = 97%*). This inconsistency may be related to differences in SSRIs types: paroxetine (36) is considered to be at risk of weight gain and deterioration of blood glucose, while sertraline and fluoxetine may be neutral or mildly beneficial. However, because this study did not distinguish between specific drugs, it was impossible to draw a clear conclusion. From the pathophysiological point of view, the hypothesis that SSRIs improve blood glucose is based on the regulation of hypothalamic-pituitary-adrenal axis and inflammatory factors by 5-HT receptors (37), but the existing evidence is weak. For diabetic patients with depression, SSRIs should not be expected to directly improve blood glucose, and hypoglycemic drugs and lifestyle interventions are still needed.

### 4.5 Quality of evidence

We assessed the certainty of evidence using the GRADE framework for each outcome (**Table 2**). The results showed that the certainty was moderate for HARS and BDI, and low for HDRS, HbA1c, FPG, PPG, and BMI. The moderate−certainty evidence (downgraded due to risk of bias) suggests that the true effect is likely close to the estimate — that is, SSRIs may be associated with a small increase in HARS scores, (MD = 2.37, 95% CI [0.73, 4.02]), while the effect on BDI remains uncertain. The low−certainty evidence (downgraded for both risk of bias and inconsistency) indicates that the true effect may be substantially different from the estimate. For HDRS, the mean difference was (MD = 0.16, 95% CI [-0.69, 1.01]), which is below the clinically meaningful threshold. For glycaemic parameters (HbA1c, FPG, PPG) and BMI, the confidence intervals included no effect, and the precision was acceptable based on the optimal information size; however, the low certainty due to bias and heterogeneity precludes firm conclusions. The main limitations were: (a) high or unclear risk of bias in most studies, especially regarding blinding and allocation concealment; (b) substantial statistical heterogeneity (*I² > 60%*) for several outcomes (HDRS, HbA1c, FPG, PPG, BMI). No serious imprecision or indirectness was identified for any outcome. Given the moderate−to−low certainty, the findings should be interpreted cautiously in clinical practice, and high−quality RCTs are needed to confirm the efficacy and safety of SSRIs in diabetic patients with depression.

**Table 2:** Certainty of evidence.

Certainty assessment							№ of patients		Effect		Certainty	Importance
№ of studies	Study design	Risk of bias	Inconsistency	Indirectness	Imprecision	Other considerations	experimental	control	Relative (95% CI)	Absolute (95% CI)		
HDRS												
10	randomised trials	seriousa	seriousb	not serious	not serious	none	418	407	–	MD 0.16 higher (0.69 lower to 1.01 higher)	⨁⨁◯◯ Lowa,b	CRITICAL
HARS												
4	randomised trials	seriousa	not serious	not serious	not serious	none	168	181	–	MD 2.37 higher (0.73 higher to 4.02 higher)	⨁⨁⨁◯ Moderatea	CRITICAL
BDI												
4	randomised trials	seriousa	not serious	not serious	not serious	none	144	110	–	MD 1.17 lower (3.69 lower to 1.35 higher)	⨁⨁⨁◯ Moderatea	CRITICAL
HbA1c												
12	randomised trials	seriousa	seriousb	not serious	not serious	none	523	476	–	MD 0.11 higher (0.06 lower to 0.28 higher)	⨁⨁◯◯ Lowa,b	IMPORTANT
FPG												
7	randomised trials	seriousa	seriousb	not serious	not serious	none	282	287	–	MD 2.55 lower (8.55 lower to 3.44 higher)	⨁⨁◯◯ Lowa,b	IMPORTANT
PPG												
4	randomised trials	seriousa	seriousb	not serious	not serious	none	170	166	–	MD 3.19 lower (15.63 lower to 9.25 higher)	⨁⨁◯◯ Lowa,b	IMPORTANT
BMI												
3	randomised trials	seriousa	seriousb	not serious	not serious	none	130	111	–	MD 0.57 higher (1.49 lower to 2.64 higher)	⨁⨁◯◯ Lowa,b	IMPORTANT

CI, confidence interval; MD, mean differenceExplanations.

^a^ Downgraded by one level for risk of bias: most included studies had high or unclear risk of bias, particularly in blinding of participants and personnel and allocation concealment (assessed using Cochrane Ro B 2 tool).

^b^ Downgraded by one level for inconsistency: substantial statistical heterogeneity was present (*I² > 60%, p < 0.05*) and the direction of effect varied across studies; No serious imprecision: the total sample size met the optimal information size or the 95% confidence interval did not cross the null and excluded clinically meaningful harm/benefit.; No serious indirectness: the populations, interventions, comparators, and outcomes were directly applicable to the review question.

### 4.6 Security issues

Because the original studies generally did not systematically report adverse events (especially the lack of quantitative data), this study could not carry out safety meta-analysis (such as suicidal ideation, hypoglycemia risk, gastrointestinal reactions, etc.). However, according to the text description of the included literature, the overall tolerance of SSRIs was acceptable, and no serious hypoglycemic events were reported. However, one study (18) suggested that insomnia and headache were more common in the sertraline group. Future research should use standardized adverse event collection tools.

### 4.7 Sources and limitations of heterogeneity

Most outcomes showed substantial heterogeneity *(I² > 80%*), mainly due to inconsistent direction of effects across studies (e.g., 24 showed opposite direction for FPG and PPG), differences in follow−up duration (< 6 months vs ≥ 6 months), mixed control types (placebo, psychotherapy, conventional treatment) with different background effects, variations in SSRI type, dose, and treatment adherence, and patient-related characteristics, including age, sex, baseline depression severity, diabetes duration (ranging from newly diagnosed to long-standing disease), and physical activity levels – all of which are known to influence antidepressant response and glycaemic control, and may therefore have confounded the pooled estimates. Specifically, HbA1c (*I² = 92%*) and BDI (*I² = 81%*) exhibited very high heterogeneity: for HbA1c, the direction of effect varied across studies – some favoured SSRIs, others favoured control; for BDI, the inconsistency was driven by two studies (Lustman et al. and Williams et al.) showing opposite directions. These high levels of heterogeneity reduce confidence in the pooled estimates and were a major reason for downgrading the GRADE certainty for these outcomes to low.

Subgroup analysis was only stratified by follow−up duration and could not be further stratified by control type or SSRI type, which is the main limitation. Additional limitations include the low methodological quality of included studies (most studies had insufficient reporting of random sequence generation, allocation concealment, and blinding; all nine studies were open−label or single−blind, and only three clearly reported allocation concealment). Although objective outcomes (HbA1c, blood glucose) are less susceptible to bias, the subjective outcomes (depression and anxiety scales) may be influenced by lack of blinding, which substantially limits the internal validity and contributed to downgrading the GRADE certainty to moderate or low for these outcomes. Furthermore, the small number of studies (only nine RCTs with 770 participants; individual sample sizes ranged from 20 to 128) and limited sample size reduced statistical power and precision, especially for secondary outcomes (HARS, PPG, BMI) reported in only 3–4 studies each. However, due to the absence of individual patient-level data, we were unable to perform subgroup analyses based on these patient-related characteristics, which remains a notable limitation of the present review. Finally, because fewer than ten studies were included, funnel plots and Egger’s test were not performed to assess publication bias, and asymmetry may exist for some indicators.

Given these limitations, our findings should be considered exploratory. Larger, well-designed double-blind RCTs with longer follow-up, uniform control conditions, standardised reporting of patient characteristics, and systematic collection of adverse events are needed to confirm the observed effects and reduce heterogeneity.

### 4.8 Clinical and research implications

Clinical practice: For patients with diabetes mellitus complicated with depression, if SSRIs are considered, patients should be informed that the antidepressant effect may take more than 6 months to fully appear, and may aggravate anxiety in the short term. It is recommended to halve the initial dose, slowly increase the dose, and monitor anxiety and activation symptoms. Do not expect SSRIs to replace hypoglycemic therapy. Future research: 1)Conducting large-scale, long-term follow-up (≥ 6 months), placebo-controlled RCTs, and pre-designating subgroups (SSRIs type, control type, baseline depression severity); 2)HDRS was used as the main depression outcome, and HARS was used as the secondary anxiety outcome; 3)Systematic collection of adverse events, especially hypoglycemia, weight gain, sexual dysfunction; 4)Explore predictive factors (such as gender, duration of diabetes, inflammatory markers) to guide individualized medication.

## 5 Conclusion

This systematic review and meta-analysis evaluated the efficacy and safety of SSRIs in diabetic patients with depression. The overall certainty of evidence was low to moderate, so the following conclusions should be interpreted as preliminary. Antidepressant efficacy depends on treatment duration: for treatment lasting less than six months, no significant benefit was observed, whereas when extended to six months or longer, SSRIs were associated with a modest but statistically significant reduction in depression scores, suggesting that an adequate course of at least six months may be necessary to achieve antidepressant effects. Regarding anxiety, SSRIs were associated with a small increase in anxiety scores, indicating a possible anxiogenic effect particularly in the early treatment phase, and clinicians should monitor anxiety symptoms closely. No significant effects were observed for any glycemic parameter (HbA1c, FPG, PPG) or for BMI, all based on low−certainty evidence. Overall, SSRIs may offer modest long−term antidepressant benefits but do not improve glycemic control or reduce weight, and they may temporarily worsen anxiety. Given the low−to−moderate certainty of evidence, these findings should be considered hypothesis−generating rather than definitive. Future high−quality RCTs with longer follow−up (≥ 6 months), clearer stratification by SSRI type, and systematic collection of adverse events are urgently needed to confirm or refute these observations.

## Data Availability

The original contributions presented in the study are included in the article/**Supplementary Material**. Further inquiries can be directed to the corresponding author.
