# Supplementary material for: Efficacy and safety of selective serotonin reuptake inhibitors for diabetes mellitus with depression: a systematic review and meta-analysis
Source: Front Endocrinol (Lausanne). 2026 Jul 22;17:1891729. doi: 10.3389/fendo.2026.1891729 (PMC13437470; doi:10.3389/fendo.2026.1891729)
Supplement: Supplementary file 2 [file Supplementaryfile2.docx]

**China National Knowledge Infrastructure(CKNI) 546**

TKA='糖尿病' and TKA='抑郁' and TKA='选择性血清素再摄取抑制剂' + '选择性5-羟色胺再吸收抑制剂' + '氟西汀' +'百优解' + '帕罗西汀' + '赛乐特' + '舍曲林' + '左洛复' + '氟伏沙明' + '兰释' + '西酞普兰' + '喜普妙' + '艾司西酞普兰' + '非莫西汀' + '吲达品' + '阿莫西平' + '氯米普明' + '芬氟拉明' + '诺芬氟拉明' + '曲唑酮' + '齐梅尔丁' + '盐酸维拉唑酮' + '奥氮平' + '沃替西汀' + '度洛西汀' + '米氮平'

**Wanfang Database 270**

题名或关键词:(糖尿病) and 题名或关键词:(抑郁) and 题名或关键词:(选择性血清素再摄取抑制剂 OR 选择性5-羟色胺再吸收抑制剂 OR 氟西汀 OR百优解 OR 帕罗西汀 OR 赛乐特 OR 舍曲林 OR 左洛复 OR 氟伏沙明 OR 兰释 OR 西酞普兰 OR 喜普妙 OR 艾司西酞普兰 OR 非莫西汀 OR 吲达品 OR 阿莫西平 OR 氯米普明 OR 芬氟拉明 OR 诺芬氟拉明 OR 曲唑酮 OR 齐梅尔丁 OR 盐酸维拉唑酮 OR 奥氮平 OR 沃替西汀 OR 度洛西汀 OR 米氮平)

Weipu Information Resource Integration Service Platform **195**

((题名或关键词=糖尿病 AND (题名或关键词=抑郁 OR 题名或关键词=情志异常)) AND (((((((((((((((((((((((((题名或关键词=选择性血清素再摄取抑制剂 OR (题名或关键词=选择性5 AND ( NOT 题名或关键词=羟色胺再吸收抑制剂))) OR 题名或关键词=氟西汀) OR 题名或关键词=百优解) OR 题名或关键词=帕罗西汀) OR 题名或关键词=赛乐特) OR 题名或关键词=舍曲林) OR 题名或关键词=左洛复) OR 题名或关键词=氟伏沙明) OR 题名或关键词=兰释) OR 题名或关键词=西酞普兰) OR 题名或关键词=喜普妙) OR 题名或关键词=艾司西酞普兰) OR 题名或关键词=非莫西汀) OR 题名或关键词=吲达品) OR 题名或关键词=阿莫西平) OR 题名或关键词=氯米普明) OR 题名或关键词=芬氟拉明) OR 题名或关键词=诺芬氟拉明) OR 题名或关键词=曲唑酮) OR 题名或关键词=齐梅尔丁) OR 题名或关键词=盐酸维拉唑酮) OR 题名或关键词=奥氮平) OR 题名或关键词=沃替西汀) OR 题名或关键词=度洛西汀) OR 题名或关键词=米氮平))

the Chinese Clinical Trial Registry (CHICCTR) 0

**PubMed 336**

"Diabetes Mellitus"[Mesh]

(Diabetes Insipidus[Title/Abstract]) OR (Diabet*[Title/Abstract]) OR (DM[Title/Abstract])

"Depression"[Mesh]

(Depressive Symptom*[Title/Abstract]) OR (Emotional Depression[Title/Abstract]) OR (Depressi* Disorder*[Title/Abstract]) OR (Depressi* Neuro*[Title/Abstract]) OR (Endogenous Depression[Title/Abstract]) OR (Melancholia[Title/Abstract]) OR (Unipolar Depression[Title/Abstract]) OR (Depressive Syndrome[Title/Abstract]) OR (mental health problems[Title/Abstract]) OR (Distress[Title/Abstract]) OR (Dysthymic disorder[Title/Abstract])

"Selective Serotonin Reuptake Inhibitors"[Mesh]

(Serotonin Uptake Inhibitors/blood[Title/Abstract]) OR (Serotonin Uptake Inhibitors/therapeutic use[Title/Abstract]) OR (5-HT Uptake Inhibitor*[Title/Abstract]) OR (5-Hydroxytryptamine Uptake Inhibitor*[Title/Abstract]) OR (ssri*[Title/Abstract]) OR (selective serotonin OR fluoxetine[Title/Abstract]) OR (Citalopram[Title/Abstract]) OR (escitalopram[Title/Abstract]) OR (Fluvoxamine[Title/Abstract]) OR (sertraline[Title/Abstract]) OR (Paroxetine[Title/Abstract]) OR (Prozac[Title/Abstract]) OR (Paxil[Title/Abstract]) OR (Zoloft[Title/Abstract]) OR (Celexa[Title/Abstract]) OR (Lexapro[Title/Abstract]) OR (femoxetine[Title/Abstract]) OR (indalpine[Title/Abstract]) OR (alaproclate[Title/Abstract]) OR (Amoxapine[Title/Abstract]) OR (Clomipramine[Title/Abstract]) OR (Fenfluramine[Title/Abstract]) OR (Norfenfluramine[Title/Abstract]) OR (Trazodone[Title/Abstract]) OR (Zimeldine[Title/Abstract]) OR (Vilazodone Hydrochloride[Title/Abstract]) OR (Olanzapine[Title/Abstract]) OR (Vortioxetine[Title/Abstract]) OR (Mirtazapine[Title/Abstract]) OR (Duloxetine[Title/Abstract])

(Random* controlled trial[Title/Abstract]) OR (Control* clinical trial[Title/Abstract]) OR (clinical trial* as topic[Title/Abstract]) OR (random allocation[Title/Abstract]) OR (double-blind method[Title/Abstract]) OR (single-blind method[Title/Abstract]) OR (research design[Title/Abstract]) OR (comparative study[Title/Abstract]) OR (evaluation studies[Title/Abstract]) OR (follow-up studies[Title/Abstract]) OR (Prospectiv* studies[Title/Abstract]) OR (cross-over studies[Title/Abstract]) OR (singl*[Title/Abstract]) OR (doubl*[Title/Abstract]) OR (trebl*[Title/Abstract]) OR (mask*[Title/Abstract]) OR (blind*[Title/Abstract]) OR (placebo*[Title/Abstract]) OR (volunteer*[Title/Abstract])

**Web of Science 193**

TS=(Diabetes Mellitus) OR AB=(Diabetes Insipidus) OR AB=(Diabet*) OR AB=(DM)

TS=(Depression) OR AB=(Depressive Symptom*) OR AB=(Emotional Depression) OR AB=(Depressi* Disorder*) OR AB=(Depressi* Neuro*) OR AB=(Endogenous Depression) OR AB=(Melancholia) OR AB=(Unipolar Depression) OR AB=(Depressive Syndrome) OR AB=(mental health problems) OR AB=(Distress) OR AB=(Dysthymic disorder)

TS=(Selective Serotonin Reuptake Inhibitors) OR AB=(Serotonin Uptake Inhibitors/blood) OR AB=(Serotonin Uptake Inhibitors/therapeutic use) OR AB=(5-HT Uptake Inhibitor*) OR AB=(5-Hydroxytryptamine Uptake Inhibitor*) OR AB=(ssri*) OR AB=(selective serotonin) OR AB=(fluoxetine) OR AB=(Citalopram) OR AB=(escitalopram) OR AB=(Fluvoxamine) OR AB=(sertraline) OR AB=(Paroxetine) OR AB=(Prozac) OR AB=(Paxil) OR AB=(Zoloft) OR AB=(Celexa) OR AB=(Lexapro) OR AB=(femoxetine) OR AB=(indalpine) OR AB=(alaproclate) OR AB=(Amoxapine) OR AB=(Clomipramine) OR AB=(Fenfluramine) OR AB=(Norfenfluramine) OR AB=(Trazodone) OR AB=(Zimeldine) OR AB=(Vilazodone Hydrochloride) OR AB=(Olanzapine) OR AB=(Vortioxetine) OR AB=(Mirtazapine) OR AB=(Duloxetine)

AB=(Random* controlled trial) OR AB=(Control* clinical trial) OR AB=(clinical trial* as topic) OR AB=(random allocation) OR AB=(double-blind method) OR AB=(single-blind method) OR AB=(research design) OR AB=(comparative study) OR AB=(evaluation studies) OR AB=(follow-up studies) OR AB=(Prospectiv* studies) OR AB=(cross-over studies) OR AB=(singl*) OR AB=(doubl*) OR AB=(trebl*) OR AB=(mask*) OR AB=(blind*) OR AB=(placebo*) OR AB=(volunteer*)

**Embase 107**

'diabetes mellitus':ab,ti OR 'diabetes insipidus':ab,ti OR 'diabet*':ab,ti OR 'DM':ab,ti

'Depressive Symptom*':ab,ti OR 'Emotional Depression':ab,ti OR 'Depressi* Disorder*':ab,ti OR 'Depressi* Neuro*':ab,ti OR 'Endogenous Depression':ab,ti OR 'Melancholia':ab,ti OR 'Unipolar Depression':ab,ti OR 'Depressive Syndrome':ab,ti OR 'mental health problems':ab,ti OR 'Distress':ab,ti OR 'Dysthymic disorder':ab,ti

'Selective Serotonin Reuptake Inhibitors':ab,ti OR 'Serotonin Uptake Inhibitors/blood':ab,ti OR 'Serotonin Uptake Inhibitors/therapeutic use':ab,ti OR '5-HT Uptake Inhibitor*':ab,ti OR '5-Hydroxytryptamine Uptake Inhibitor*':ab,ti OR 'ssri*':ab,ti OR 'selective serotonin OR fluoxetine':ab,ti OR 'Citalopram':ab,ti OR 'escitalopram':ab,ti OR 'Fluvoxamine':ab,ti OR 'sertraline':ab,ti OR 'Paroxetine':ab,ti OR 'Prozac':ab,ti OR 'Paxil':ab,ti OR 'Zoloft':ab,ti OR 'Celexa':ab,ti OR 'Lexapro':ab,ti OR 'femoxetine':ab,ti OR 'indalpine':ab,ti OR 'alaproclate':ab,ti OR 'Amoxapine':ab,ti OR 'Clomipramine':ab,ti OR 'Fenfluramine':ab,ti OR 'Norfenfluramine':ab,ti OR 'Trazodone':ab,ti OR 'Zimeldine':ab,ti OR 'Vilazodone Hydrochloride':ab,ti OR 'Olanzapine':ab,ti OR 'Vortioxetine':ab,ti OR 'Mirtazapine':ab,ti OR 'Duloxetine':ab,ti

'Random* controlled trial':ab,ti OR 'Control* clinical trial':ab,ti OR 'clinical trial* as topic':ab,ti OR 'random allocation':ab,ti OR 'double-blind method':ab,ti OR 'single-blind method':ab,ti OR 'research design':ab,ti OR 'comparative study':ab,ti OR 'evaluation studies':ab,ti OR 'follow-up studies':ab,ti OR 'Prospectiv* studies':ab,ti OR 'cross-over studies':ab,ti OR 'singl*':ab,ti OR 'doubl*':ab,ti OR 'trebl*':ab,ti OR 'mask*':ab,ti OR 'blind*':ab,ti OR 'placebo*':ab,ti OR 'volunteer*':ab,ti

**Scopus 1895**

(TITLE-ABS-KEY(Diabetes Mellitus) OR TITLE-ABS-KEY(Diabetes Insipidus) OR TITLE-ABS-KEY(Diabet*) OR TITLE-ABS-KEY(DM)) AND (TITLE-ABS-KEY(Depression) OR TITLE-ABS-KEY(Depressive Symptom*) OR TITLE-ABS-KEY(Emotional Depression) OR TITLE-ABS-KEY(Depressi* Disorder*) OR TITLE-ABS-KEY(Depressi* Neuro*) OR TITLE-ABS-KEY(Endogenous Depression) OR TITLE-ABS-KEY(Melancholia) OR TITLE-ABS-KEY(Unipolar Depression) OR TITLE-ABS-KEY(Depressive Syndrome) OR TITLE-ABS-KEY(mental health problems) OR TITLE-ABS-KEY(Distress) OR TITLE-ABS-KEY(Dysthymic disorder)) AND (TITLE-ABS-KEY(Selective Serotonin Reuptake Inhibitors) OR TITLE-ABS-KEY(Serotonin Uptake Inhibitors/blood) OR TITLE-ABS-KEY(Serotonin Uptake Inhibitors/therapeutic use) OR TITLE-ABS-KEY(5-HT Uptake Inhibitor*) OR TITLE-ABS-KEY(5-Hydroxytryptamine Uptake Inhibitor*) OR TITLE-ABS-KEY(ssri*) OR TITLE-ABS-KEY(selective serotonin OR fluoxetine) OR TITLE-ABS-KEY(Citalopram) OR TITLE-ABS-KEY(escitalopram) OR TITLE-ABS-KEY(Fluvoxamine) OR TITLE-ABS-KEY(sertraline) OR TITLE-ABS-KEY(Paroxetine) OR TITLE-ABS-KEY(Prozac) OR TITLE-ABS-KEY(Paxil) OR TITLE-ABS-KEY(Zoloft) OR TITLE-ABS-KEY(Celexa) OR TITLE-ABS-KEY(Lexapro) OR TITLE-ABS-KEY(femoxetine) OR TITLE-ABS-KEY(indalpine) OR TITLE-ABS-KEY(alaproclate) OR TITLE-ABS-KEY(Amoxapine) OR TITLE-ABS-KEY(Clomipramine) OR TITLE-ABS-KEY(Fenfluramine) OR TITLE-ABS-KEY(Norfenfluramine) OR TITLE-ABS-KEY(Trazodone) OR TITLE-ABS-KEY(Zimeldine) OR TITLE-ABS-KEY(Vilazodone Hydrochloride) OR TITLE-ABS-KEY(Olanzapine) OR TITLE-ABS-KEY(Vortioxetine) OR TITLE-ABS-KEY(Mirtazapine) OR TITLE-ABS-KEY(Duloxetine)) AND (TITLE-ABS-KEY(Random* controlled trial) OR TITLE-ABS-KEY(Control* clinical trial) OR TITLE-ABS-KEY(clinical trial* as topic) OR TITLE-ABS-KEY(random allocation) OR TITLE-ABS-KEY(double-blind method) OR TITLE-ABS-KEY(single-blind method) OR TITLE-ABS-KEY(research design) OR TITLE-ABS-KEY(comparative study) OR TITLE-ABS-KEY(evaluation studies) OR TITLE-ABS-KEY(follow-up studies) OR TITLE-ABS-KEY(Prospectiv* studies) OR TITLE-ABS-KEY(cross-over studies) OR TITLE-ABS-KEY(singl*) OR TITLE-ABS-KEY(doubl*) OR TITLE-ABS-KEY(trebl*) OR TITLE-ABS-KEY(mask*) OR TITLE-ABS-KEY(blind*) OR TITLE-ABS-KEY(placebo*) OR TITLE-ABS-KEY(volunteer*))

**Cochrane Library 267**

(Diabetes Mellitus):ti,ab,kw OR (Diabetes Insipidus):ti,ab,kw OR (Diabet*):ti,ab,kw OR (DM):ti,ab,kw

(Depression):ti,ab,kw OR (Depressive Symptom*):ti,ab,kw OR (Emotional Depression):ti,ab,kw OR (Depressi* Disorder*):ti,ab,kw OR (Depressi* Neuro*):ti,ab,kw OR (Endogenous Depression):ti,ab,kw OR (Melancholia):ti,ab,kw OR (Unipolar Depression):ti,ab,kw OR (Depressive Syndrome):ti,ab,kw OR (mental health problems):ti,ab,kw OR (Distress):ti,ab,kw OR (Dysthymic disorder):ti,ab,kw

(Selective Serotonin Reuptake Inhibitors):ti,ab,kw OR (Serotonin Uptake Inhibitors):ti,ab,kw OR (Serotonin Uptake Inhibitors):ti,ab,kw OR (5HT Uptake Inhibitor*):ti,ab,kw OR (5Hydroxytryptamine Uptake Inhibitor*):ti,ab,kw OR (ssri*):ti,ab,kw OR (selective serotonin OR fluoxetine):ti,ab,kw OR (Citalopram):ti,ab,kw OR (escitalopram):ti,ab,kw OR (Fluvoxamine):ti,ab,kw OR (sertraline):ti,ab,kw OR (Paroxetine):ti,ab,kw OR (Prozac):ti,ab,kw OR (Paxil):ti,ab,kw OR (Zoloft):ti,ab,kw OR (Celexa):ti,ab,kw OR (Lexapro):ti,ab,kw OR (femoxetine):ti,ab,kw OR (indalpine):ti,ab,kw OR (alaproclate):ti,ab,kw OR (Amoxapine):ti,ab,kw OR (Clomipramine):ti,ab,kw OR (Fenfluramine):ti,ab,kw OR (Norfenfluramine):ti,ab,kw OR (Trazodone):ti,ab,kw OR (Zimeldine):ti,ab,kw OR (Vilazodone Hydrochloride):ti,ab,kw OR (Olanzapine):ti,ab,kw OR (Vortioxetine):ti,ab,kw OR (Mirtazapine):ti,ab,kw OR (Duloxetine):ti,ab,kw

(Random* controlled trial):ti,ab,kw OR (Control* clinical trial):ti,ab,kw OR (clinical trial* as topic):ti,ab,kw OR (random allocation):ti,ab,kw OR (double-blind method):ti,ab,kw OR (single-blind method):ti,ab,kw OR (research design):ti,ab,kw OR (comparative study):ti,ab,kw OR (evaluation studies):ti,ab,kw OR (follow-up studies):ti,ab,kw OR (Prospectiv* studies):ti,ab,kw OR (cross-over studies):ti,ab,kw OR (singl*):ti,ab,kw OR (doubl*):ti,ab,kw OR (trebl*):ti,ab,kw OR (mask*):ti,ab,kw OR (blind*):ti,ab,kw OR (placebo*):ti,ab,kw OR (volunteer*):ti,ab,kw
